# 8-Gingerol Ameliorates Myocardial Fibrosis by Attenuating Reactive Oxygen Species, Apoptosis, and Autophagy *via* the PI3K/Akt/mTOR Signaling Pathway

**DOI:** 10.3389/fphar.2021.711701

**Published:** 2021-07-28

**Authors:** Yucong Xue, Muqing Zhang, Miaomiao Liu, Yu Liu, Li Li, Xue Han, Zhenqing Sun, Li Chu

**Affiliations:** ^1^ College of Integrative Medicine, Hebei University of Chinese Medicine, Shijiazhuang, China; ^2^ Affiliated Hospital, Hebei University of Chinese Medicine, Shijiazhuang, China; ^3^ School of Pharmacy, Hebei University of Chinese Medicine, Shijiazhuang, China; ^4^ School of Pharmacy, Hebei Medical University, Shijiazhuang, China; ^5^ Hebei Higher Education Institute Applied Technology Research Center on TCM Formula Preparation, Shijiazhuang, China; ^6^ Qingdao Hospital of Traditional Chinese Medicine, Qingdao Hiser Hospital, Qingdao, China; ^7^ Hebei Key Laboratory of Chinese Medicine Research on Cardio-cerebrovascular Disease, Shijiazhuang, China

**Keywords:** 8-gingerol, myocardial fibrosis, reactive oxygen species, apoptosis, autophagy, PI3K/Akt/mTOR signaling pathway

## Abstract

8-gingerol (8-Gin) is the series of phenolic substance that is extracted from ginger. Although many studies have revealed that 8-Gin has multiple pharmacological properties, the possible underlying mechanisms of 8-Gin against myocardial fibrosis (MF) remains unclear. The study examined the exact role and potential mechanisms of 8-Gin against isoproterenol (ISO)-induced MF. Male mice were intraperitoneally injected with 8-Gin (10 and 20 mg/kg/d) and concurrently subcutaneously injected with ISO (10 mg/kg/d) for 2 weeks. Electrocardiography, pathological heart morphology, myocardial enzymes, reactive oxygen species (ROS) generation, degree of apoptosis, and autophagy pathway-related proteins were measured. Our study observed 8-Gin significantly reduced J-point elevation and heart rate. Besides, 8-Gin caused a marked decrease in cardiac weight index and left ventricle weight index, serum levels of creatine kinase and lactate dehydrogenase (CK and LDH, respectively), ROS generation, and attenuated ISO-induced pathological heart damage. Moreover, treatment with 8-Gin resulted in a marked decrease in the levels of collagen types I and III and TGF-β in the heart tissue. Our results showed 8-Gin exposure significantly suppressed ISO-induced autophagosome formation. 8-Gin also could lead to down-regulation of the activities of matrix metalloproteinases-9 (MMP-9), Caspase-9, and Bax protein, up-regulation of the activity of Bcl-2 protein, and alleviation of cardiomyocyte apoptosis. Furthermore, 8-Gin produced an obvious increase in the expressions of the PI3K/Akt/mTOR signaling pathway-related proteins. Our data showed that 8-Gin exerted cardioprotective effects on ISO-induced MF, which possibly occurred in connection with inhibition of ROS generation, apoptosis, and autophagy *via* modulation of the PI3K/Akt/mTOR signaling pathway.

## Introduction

Myocardial fibrosis (MF) refers to excessive accumulation of collagen fibers and a significant increase in collagen concentration in the myocardium (Lu et al., 2018). Accumulating research has demonstrated that MF exists in many cardiovascular diseases, which is closely related to arrhythmia, cardiac dysfunction, and sudden cardiac death (Kong et al., 2014; Bittencourt et al., 2019; Piek et al., 2019). With the increasing morbidity of cardiovascular diseases worldwide, a large number of researchers are paying more and more attention to MF. However, due to the relatively complex pathogenesis and molecular mechanisms, there is still a lack of effective therapies that can halt or reverse MF.

Previous studies have found that reactive oxygen species (ROS) induced the initiation of fibrosis through secretion of multifarious profibrotic factors (Schirone et al., 2017; Zhang et al., 2018; Kang et al., 2019). In recent years, an increasing number of studies on autophagy in cardiovascular diseases can be found, and researchers have also gradually realized the special relationship between autophagy and MF. Autophagy, a lysosome-dependent degradation pathway, exists widely in eukaryotes (Tayebjee et al., 2003). Autophagy is a mechanism of cell self-protection, which is essential for maintaining the homeostasis of the intracellular environment (Li et al., 2018a). Under normal physiological conditions, basal levels of autophagy activity in the heart occur. However, abundant studies have reported that nutrient deprivation (Ryan et al., 2007), oxidative stress (Medugorac and Jacob, 1983), hypoxia (de Jong et al., 2011), and ischemia-reperfusion (Chiao et al., 2012) can stimulate cell autophagy. Excessive autophagy results in autophagic cell death. Many researchers have found that reactive oxygen species (ROS) form one of the main intracellular signal transducers in the autophagy process (Cabral-Pacheco et al., 2020). Excessive generation of ROS causes oxidative damage in the myocardium and then leads to the hyperactivation of autophagy followed by cardiomyocyte apoptosis (Li et al., 2014). Apoptosis is an autonomous cell death process regulated by Bcl-2 and Caspase family proteins. Apoptosis is essential for maintaining cell homeostasis. However, oxygen deficiency occurrence leads to the excessive production of ROS, causes up-regulation of the expression of Bax followed by release of cytochrome c, and then cleaves Caspase-9 leading to activation of the mitochondrial apoptosis pathway, thus regulating the process of apoptosis (Gatica et al., 2015; Hu et al., 2015).

The regulation of autophagy is relatively complex. The most classical signaling pathway involved in the regulation of autophagy is the phosphatidylinositol-3-kinase/protein kinase B/mammalian target of rapamycin (PI3K/Akt/mTOR) signaling pathway (Wu et al., 2021). Previous research has indicated that the PI3K/Akt/mTOR signaling pathway is involved in the regulation of cell proliferation, metabolism, growth, differentiation, and apoptosis (Tang et al., 2015). More importantly, the PI3K/Akt/mTOR signaling pathway plays an essential role in the pathogenesis of cardiovascular diseases *via* the modulation of autophagy (Zhang et al., 2017a). Activation of the PI3K/AKT signaling pathway induces AKT protein activation, and the phosphorylation of AKT protein (p-AKT) inhibits the protein expression of Caspase-9 and Bax (Eguchi et al., 2012; Filomeni et al., 2015). The kinase mTOR has always been considered a key negative autophagy regulator downstream of the PI3K/AKT signaling pathway. mTOR can be phosphorylated (p-mTOR) by p-Akt (Dadakhujaev et al., 2009), and p-mTOR can inhibit autophagy scavenging ubiquitin and induce phosphorylation inactivation of Beclin1 protein (Jiang et al., 2017a).

**FIGURE 1:**
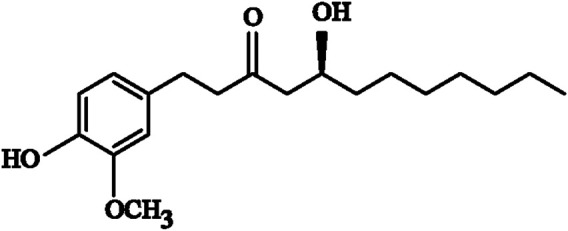
Molecular structural formula of 8-gingerol (8-Gin).

Ginger is the fresh root of *Zingiber officinale Roscoe* (Zingiberaceae), which has an aromatic and pungent taste. Ginger has been widely used as a dietary supplement worldwide (Zhao et al., 2020). Gingerol is a type of phenolic constituent that is extracted from ginger, and is not only the main pungent active ingredient but also one of the main functional substances of ginger. The health benefits of ginger are mainly attributed to gingerol (Hale et al., 2013). Due to the different chain-lengths in its molecular structure, gingerol can be subdivided into 6-, 8- and 10-gingerols (6-, 8-, and 10-Gin) (Wymann et al., 2003). Previous research has confirmed that 8-Gin (Figure 1) has various pharmacological properties, including anti-inflammatory and anti-oxidative capacities, immunosuppressive activity, and cardiotonic action (Li et al., 2018b; Takino et al., 2019; Q.; Zhao et al., 2019). However, the cardioprotective effects of 8-Gin against isoproterenol (ISO)-induced MF have not yet been reported.

Our previous study demonstrated that 6-Gin exerts an anti-MF effect (Zhang et al., 2019). Because 8-Gin and 6-Gin have similar chemical structures, we speculate that 8-Gin may also be protective against ISO-induced MF. Therefore, in order to explore the underlying mechanisms of 8-Gin against ISO-induced MF in mice, we evaluated the functions of 8-Gin on oxidative stress, apoptosis, autophagy, and the PI3K/Akt/mTOR signaling pathway.

## Materials and Methods

### Chemical Reagents

Eight-Gin was bought from Chengdu Biopurify Phytochemicals Ltd. (Chengdu, China). ISO was bought from Cayman Chemicals (Ann Arbor, Michigan, United States). Captopril (CAP) was bought from Shanghai Yuan-ye Biotechnology Co., Ltd. 8-Gin, ISO, and CAP were both dissolved in 0.9% saline and used immediately after preparation.

### Animal Experimental Procedures

Fifty male Kunming mice (aged 6–8 weeks; weight, 15–20 g) were purchased from Liaoning Changsheng Biotechnology Co., Ltd. [Liaoning, China; Certificate No. SCXK (Liaoning) 2020–0001]. All mice were bred in cages under temperature- and humidity-controlled conditions [20–22°C and 45–55% relative humidity (RH), respectively] with a 12 h light–dark cycle. All mice were given diet and water ad libitum and allowed a one-week recovery period before experiments were started.

A total of 50 mice were randomized into five groups of ten animals each: 1) control (CON), 2) ISO alone (ISO), 3) low-dose 8-Gin + ISO (8-Gin_L_ + ISO, 10 mg/kg/d), 4) high-dose 8-Gin + ISO (8-Gin_H_ + ISO, 20 mg/kg/d), and 5) captopril + ISO (CAP + ISO, 45 mg/kg/d). The MF model was established as previously described (Zhang et al., 2019; Chen et al., 2020). The CON group was given normal saline. The ISO group was subcutaneous injection with ISO (10 mg/kg/d, s.c.). The 8-Gin_L_ and 8-Gin_H_ groups were treated with 8-Gin [10 and 20 mg/kg/d, intraperitoneally (i.p.), respectively] (Nieuwenhuiset al., 2010; Zhao et al., 2019), and then subcutaneously injected with ISO (10 mg/kg/d). The CAP group was treated with CAP (45 mg/kg/d, i.p.) (Ekor, 2014), and then subcutaneously injected with ISO (10 mg/kg/d). The measurement period lasted for 14 consecutive days. No mice died during our experiment. The animals were weighed at the end of the experiments. After the experiments, all mice were anesthetized with sodium urethane (1 g/kg, i.p.), and the heart was removed and detected as described below.

### Determination of Cardiac Function

The RM6240BD Biological Signal Collection System (Chengdu, China) was used to record the electrocardiogram (ECG) of all mice after which we collected data and analyzed the changes in heart rate and J-point elevation. After the ECG measurements, the heart was quickly removed and washed in normal saline. We weighed the whole heart and then isolated left ventricle and weighed it again. Then we recorded the data and calculated both the cardiac weight index and left ventricle weight index (CWI and LVWI, respectively). The CWI was considered the ratio of the heart weight (HW) expressed as mg to the body weight (BW) expressed as g, and the LVWI was the ratio of the LVW (mg) to the BW (g).

### Determination of Creatine Kinase and Lactate Dehydrogenase

The mice blood was collected and then separated the serum by centrifugation (3,500 rpm, 10 min). According to the manufacturer’s protocols, the levels of serum CK (Catalog: A032) and LDH (Catalog: A020-2) were measured by the corresponding assay kits (Nanjing Jiancheng Bioengineering Institute, Nanjing, China).

### Histological Examination

The heart samples obtained from mice were fixed in 4% paraformaldehyde at room temperature for 48 h. After fixation, the tissue was embedded in paraffin and 4 μm thick sections were prepared. The sections were then stained with hematoxylin and eosin (H&E) or Sirius red. The results were evaluated using a Leica DM4000B light microscopy (Solms, Germany). The distribution of collagen fibers in mice hearts was observed with Sirius red staining.

### Enzyme-Linked Immunosorbent Assay

The heart tissue was ground with a homogenate medium and centrifuged at 3,500 rpm for 10 min. The supernatant was stored at 4°C until used for measurements. According to the manufacturer’s instructions, the contents of collagen type I and III and transforming growth factor-β (TGF-β) (Catalog: H142-1-1; Catalog: H143-1-1; Catalog: H034-1, respectively) were determined using the corresponding assay kits (Nanjing Jiancheng Bioengineering Institute, Nanjing, China).

### Determination of Reactive Oxygen Species

The slides were placed into a spontaneous fluorescence quenching reagent (Catalog: G1221, Servicebio technology Co., Ltd., Wuhan, China) for 5 min and washed in running tap water for 10 min. ROS staining solution was then added to the slides (Catalog: D7008, SIGMA, St. Louis, MO, United States), which were incubated at 37°C for 30 min in the dark. The slides were then incubated with 4’,6-diamidino-2 phenylindole (DAPI) solution (Catalog: G1012, Servicebio technology Co., Ltd., Wuhan, China) at room temperature for 10 min in the dark. After counterstaining, the slides were washed with phosphate-buffered saline (PBS) three times for 5 min per wash. The slides were seal-capped with mounting medium (Catalog: G1401, Servicebio technology Co., Ltd., Wuhan, China). Finally, we detected and collected images by fluorescent microscopy (Nikon Eclipse C1, Nikon, Japan).

### TdT-Mediated dUTP Nick-End Labeling Staining

The sections were deparaffinized and rehydrated. We added proteinase K working solution (Catalog: G1205, Servicebio technology Co., Ltd., Wuhan, China) to cover the tissue and incubated the sections at 37°C for 20 min. The sections were then washed three times with PBS. The slices were then immersed in 3% hydrogen peroxide (H_2_O_2_) and incubated for 20 min at room temperature in the dark after which the slices were washed three times with PBS for 5 min per wash. After the slices were slightly dried, the TdT-mediated dUTP nick-end labeling (TUNEL) reaction solution (Catalog: G1507, Servicebio technology Co., Ltd., Wuhan, China) was added to the slices and incubated at 37°C for 1 h. After washing with PBS, freshly prepared 3,3′-diaminobenzidine (DAB) chromogenic reagent was load onto the slices (Catalog: G1212, Servicebio technology Co., Ltd., Wuhan, China) and terminated the developing reaction until the nucleus showed brown-yellow. The slices were then counterstained with a hematoxylin staining solution (Catalog: G1004, Servicebio technology Co., Ltd., Wuhan, China) for 1 min, treated with the differentiating solution (Catalog: G1039, Servicebio technology Co., Ltd., Wuhan, China) for a few seconds, and finally incubated with a bluing solution (Catalog: G1040, Servicebio technology Co., Ltd., Wuhan, China). Finally, the slices were dehydrated and sealed with neutral gum. The results were evaluated through a Leica DM4000B light microscopy (Solms, Germany). The positive area was quantitatively calculated by Image-Pro Plus 6.0 software (Media Cybernetics, Bethesda, United States).

### Transmission Electron Microscope

The fresh tissues were fixed in a transmission electron microscopy (TEM) fixative (Catalog: G1102, Servicebio technology Co., Ltd., Wuhan, China) and then post-fixed with 1% osmic acid for 2 h at room temperature in the dark. The tissues were then rinsed three times and dehydrated at room temperature. Next, resin was allowed to penetrate the tissue after which the tissue was embedded. The embedding tissue was polymerized 48 h at 60°C. The samples were sectioned into 60–80 nm thick pieces and stained with 2% uranium acetate saturated alcohol solution in the dark for 8 min, washed three times, and then dried overnight at room temperature. The sections were observed under TEM (HT7800, Hitachi, Japan), and images were obtained.

### Western Blot Analysis

The frozen heart tissue was washed with cold PBS three times after which the RIPA lysis buffer (Catalog: G2002, Servicebio technology Co., Ltd., Wuhan, China) was added for homogenization. After completely homogenizing the tissue, it was centrifuged at 12,000 rpm for 10 min at 4°C, and protein concentrations from all samples were quantified with the Bicinchoninic acid (BCA) kit (Catalog: G2026, Servicebio technology Co., Ltd., Wuhan, China). The protein samples were then loaded onto a sodium dodecyl sulfate-polyacrylamide gel for electrophoresis (SDS-PAGE) and then transferred onto polyvinylidene difluoride (PVDF) membranes (Catalog: G6015, Servicebio technology Co., Ltd., Wuhan, China) for wetern blotting. After blocking with tris-buffered saline tween-20 (TBST) buffer containing skim milk for 30 min, the membranes were incubated with anti-PI3K (Catalog: 67071-1-lg, Proteintech Group Inc., Wuhan, China, 1:1,000 dilution), anti-p-PI3K (Catalog: BS-6417R, BIOSS Inc., Beijing, China, 1:1,000 dilution), anti-AKT (Catalog: GB11689, Servicebio technology Co., Ltd., Wuhan, China, 1:1,000 dilution), anti-p-AKT (Catalog: AF0832, Affinity Biosciences technology Co., Ltd., Jiangsu, China, 1:1,000 dilution), anti-mTOR (Catalog: GB111839, Servicebio technology Co., Ltd., Wuhan, China, 1:1,000 dilution), anti-p-mTOR (Catalog:BS-3495R, Catalog: BS-6417R, BIOSS Inc., Beijing, China, 1:1,000 dilution), anti-MMP-9 (Catalog: GB12132-1, Servicebio technology Co., Ltd., Wuhan, China, 1:1,000 dilution), anti-Bcl-2 (Catalog: PAA778Mu01, Cloud-Clone corp. Inc., Wuhan, China, 1:1,000 dilution), anti-Bax (Catalog: GB11690, Servicebio technology Co., Ltd., Wuhan, China, 1:1,000 dilution), anti-Caspase-9 (Catalog: GB11053-1, Servicebio technology Co., Ltd., Wuhan, China, 1:1,000 dilution), and anti-GAPDH (Catalog: GB12002, Servicebio technology Co., Ltd., Wuhan, China, 1:1,000 dilution). After incubation, we added horseradish peroxidase (HRP)-conjugated secondary antibody (Servicebio technology Co., Ltd., Wuhan, China, 1:3,000 dilution) onto the membranes for 30 min at room temperature. Finally, the blots were scanned using V370 (EPSON, Japan), and the bands’ gray values were analyzed using the AlphaEaseFC software (Alpha Innotech, United States).

### Statistical Analysis

The statistical data were expressed as the mean ± standard error of the mean (SEM). Multiple groups were compared using one-way analysis of variance (ANOVA) with Bonferroni’s test. The results were analyzed using Origin 9.1 (OriginLab Corp., Northampton, MA) software. All probability values (*p* < 0.05) were considered to be the criterion for statistical significance.

## Results

### Effects of 8-Gingerol on Gross Examination and Cardiac Function

**FIGURE 2:**
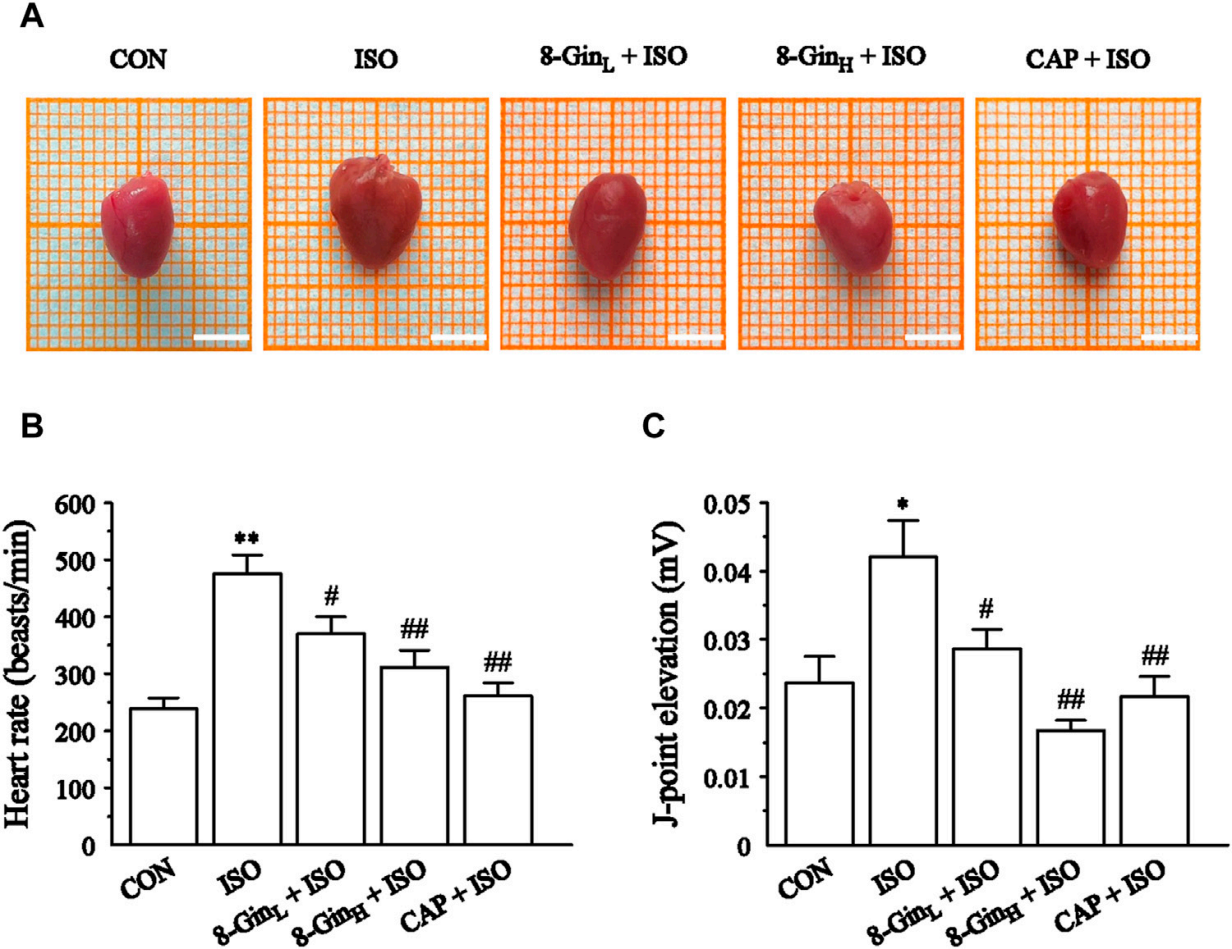
Effects of 8-Gin on gross examination and cardiac function in mice heart. **(A)** Representative photos of mice hearts in five groups. Scale bar = 0.5 cm. **(B, C)** The heart rate and J-point elevation were recorded by electrocardiogram (ECG). Data are shown as the mean ± SEM, *n* = 10. ^*^ *p* < 0.05, ^**^ *p* < 0.01 vs. the CON group; ^#^ *p* < 0.05, ^##^ *p* < 0.01 vs. the ISO group.

In Figure 2A, macroscopic observations showed a significant increase in mice heart size and a deeper color after continuous injection of ISO for 14 days compared to the CON group. However, in this same group, treatment with 8-Gin and CAP caused a marked reduction in heart size and color that was consistent with the CON group. In addition, we found the suppression of the heart rate and J-point based on ECG tracings in the 8-Gin_L_ + ISO, 8-Gin_H_ + ISO, and CAP + ISO groups (Figures 2B,C).

### Effects of 8-Gingerol on Cardiac Weight Index and Left Ventricle Weight Index

We weighed the whole hearts and left ventricles of mice to calculate the CWI and LVWI. As shown in Table 1, the HW and LVW were both clearly larger in the ISO group compared to the CON group (*p* < 0.01). In contrast, pretreatment with 8-Gin and CAP led to a decrease in the HW and LVW of mice (*p* < 0.01 or 0.05). Similarly, in the ISO group, the CWI and LVWI were distinctly increased in contrast with the CON group (*p* < 0.01), while pretreatment with 8-Gin and CAP reduced these values (*p* < 0.01 or 0.05).

**TABLE 1:** Effects of 8-Gin on CWI and LVWI.

Group	BW (g)	HW (mg)	LVW (mg)	CWI (mg/g)	LVWI (mg/g)
CON	29.31 ± 0.63	111.6 ± 6.15	62.05 ± 5.89	3.82 ± 0.22	2.12 ± 0.19
ISO	28.02 ± 0.79	156.77 ± 5.16^**^	102.50 ± 5.41^**^	5.62 ± 0.22^**^	3.67 ± 0.20^**^
8-Gin_L_ + ISO	27.29 ± 0.47	134.32 ± 4.13^##^	80.46 ± 5.80^#^	4.93 ± 0.15^#^	2.95 ± 0.21^#^
8-Gin_H_ + ISO	26.08 ± 0.71	125.21 ± 4.23^##^	72.08 ± 6.78^##^	4.81 ± 0.12^##^	2.76 ± 0.23^##^
CAP + ISO	25.85 ± 0.84	121.14 ± 4.96^##^	70.02 ± 4.41^##^	4.71 ± 0.19^##^	2.72 ± 0.17^##^

Data are shown as the mean ± SEM. ^**^ *p* < 0.01 vs. the CON group; ^#^ *p* < 0.05, ^##^ *p* < 0.01 vs. the ISO group.

### Effects of 8-Gingerol on Creatine Kinase, Lactate Dehydrogenase, and Histopathology

**FIGURE 3:**
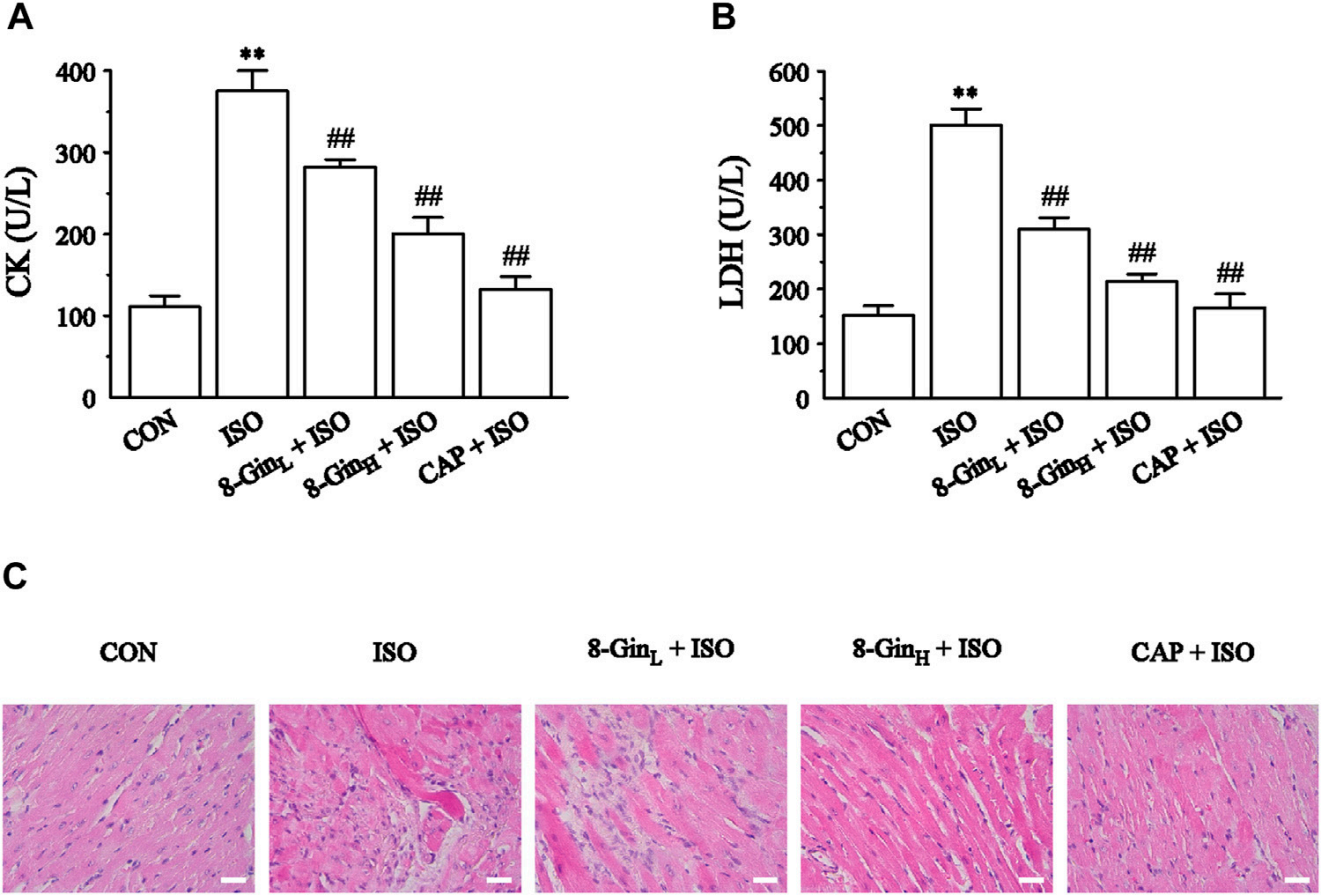
Effects of 8-Gin on cardiac biomarkers and histopathology. **(A, B)** The concentration of mice serum CK and LDH, respectively. Data are shown as the mean ± SEM, *n* = 6. ^**^ *p* < 0.01 vs. the CON group; ^##^ *p* < 0.01 vs. the ISO group. **(C)** Representative H&E staining histologic slices in five groups of CON, ISO, 8-Gin_L_ + ISO, 8-Gin_H_ + ISO, and CAP + ISO (magnification × 400, scale bar = 50 μm).

The CK and LDH activities in mice serum were measured in order to pinpoint myocardial damage. In Figures 3A,B, 8-Gin and CAP markedly depressed the levels of CK and LDH compared with the ISO group (*p* < 0.01), indicating that 8-Gin and CAP could facilitate improvements in ISO-induced myocardial damage. Also, the results of H&E staining showed interstitial edema, hyperemia, myocardial fiber thickening, and cardiomyocytes eosinophilic enhancement in the ISO group. Endomyocardial fibrosis was more serious than in the epicardium (Figure 3C). However, 8-Gin and CAP could cause an improvement in this pathological change, suggesting 8-Gin and CAP can protect against ISO-induced MF.

### Effects of 8-Gingerol on Sirius Red Staining, Transforming Growth Factor-β, and Collagen Types I and III

**FIGURE 4:**
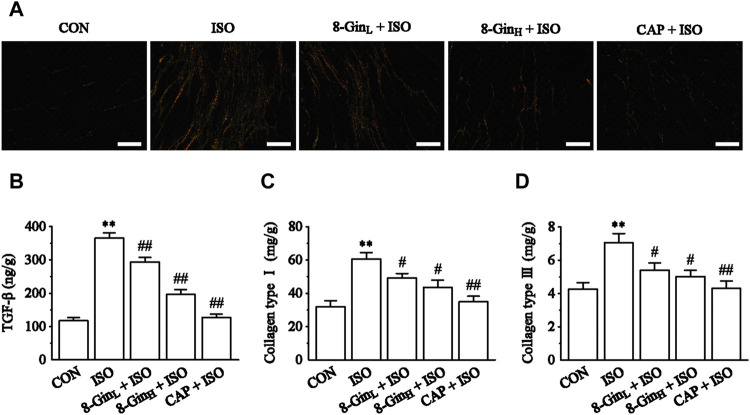
Effects of 8-Gin on Sirius red staining and collagen types I and III. **(A)** Representative Sirius red staining histologic slices in five groups of CON, ISO, 8-Gin_L_ + ISO, 8-Gin_H_ + ISO, and CAP + ISO (magnification × 200, scale bar = 100 μm). **(B–D)** Quantification analysis of TGF-β, collagen types I and III using ELISA method. Data are shown as the mean ± SEM, *n* = 6. ^**^ *p* < 0.01 vs. the CON group; ^#^ *p* < 0.05, ^##^ *p* < 0.01 vs. the ISO group.

As shown in Figure 4A, Sirius red staining analysis of cardiac tissue in the ISO group indicated heavy collagen deposition compared with the CON group. 8-Gin and CAP caused a significant decrease in collagen accumulation. In Figures 4B–D, quantitative analysis [enzyme-linked immunosorbent analysis (ELISA)] of TGF-β, and collagen type I and III contents revealed that ISO induced an increase relative to the CON group (*p* < 0.01). Compared to the ISO group, the concentrations of TGF-β, and collagen types I and III were apparently reduced in the 8-Gin_L_ + ISO, 8-Gin_H_ + ISO, and CAP + ISO groups (*p* < 0.01 or 0.05).

### Effects of 8-Gingerol on Reactive Oxygen Species Generation

**FIGURE 5:**
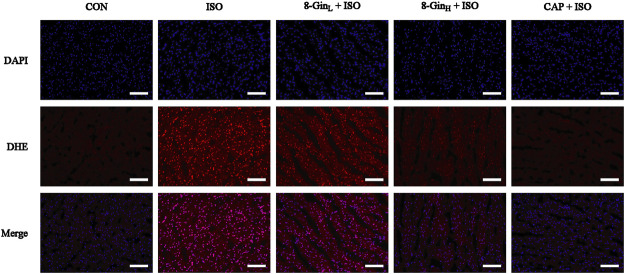
Effects of 8-Gin on ROS generation in mice heart tissue. Generation of ROS was measured by a dihydroethidium (DHE) probe staining of mice heart in CON, ISO, 8-Gin_L_ + ISO, 8-Gin_H_ + ISO, and CAP + ISO groups (magnification × 200, scale bar = 100 μm).

To evaluate the effect of 8-Gin on ROS generation, we measured the fluorescence intensity of ROS in the myocardial tissue of each group using a dihyroethidium (DHE) probe. In Figure 5, compared to the CON group, the fluorescence intensity of ROS was markedly strengthened in the ISO group. However, 8-Gin and CAP could distinctly attenuate fluorescence intensity, indicating 8-Gin and CAP could reduce ROS production.

### Effects of 8-Gingerol on TdT-Mediated dUTP Nick-End Labeling Staining Analysis

**FIGURE 6:**
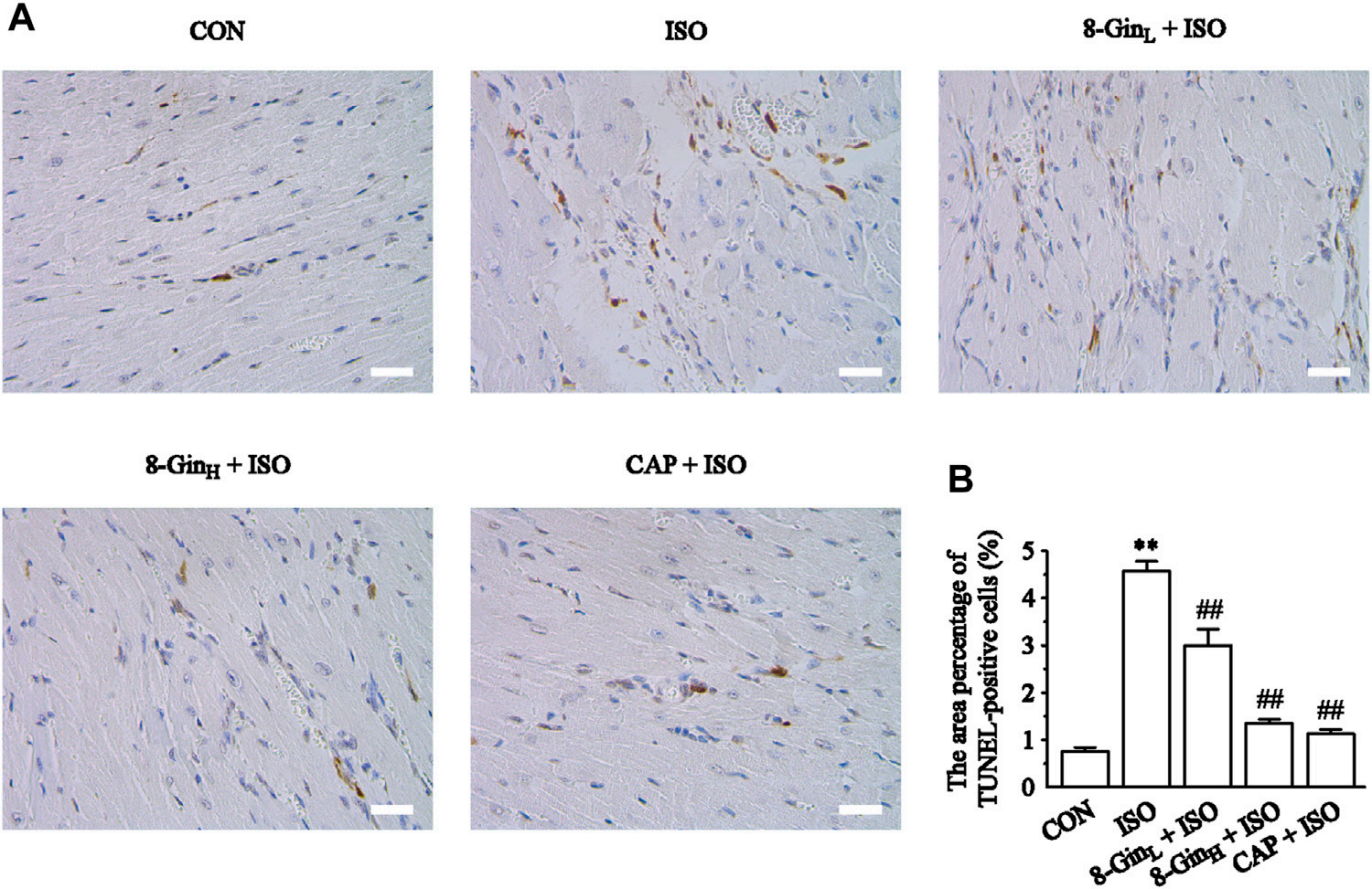
Effects of 8-Gin on TUNEL staining. **(A)** Representative TUNEL staining of cardiac sections from each group (magnification × 400, scale bar = 50 μm). **(B)** The area percentages of TUNEL-positive cells were shown. Data are shown as the mean ± SEM, *n* = 5. ^**^ *p* < 0.01 vs. the CON group; ^##^ *p* < 0.01 vs. the ISO group.

Figure 6 showed the data based on TUNEL staining in five different groups. The area percentage of TUNEL-positive cells was also calculated. According to the results, it was found that the area percentage of TUNEL-positive cells in the ISO group was larger than the CON group (*p* < 0.01). After pretreatment with 8-Gin and CAP, an obvious decrease between the 8-Gin group and ISO group occurred (*p* < 0.01).

### Effects of 8-Gingerol on the Formation of Autophagosomes

**FIGURE 7:**
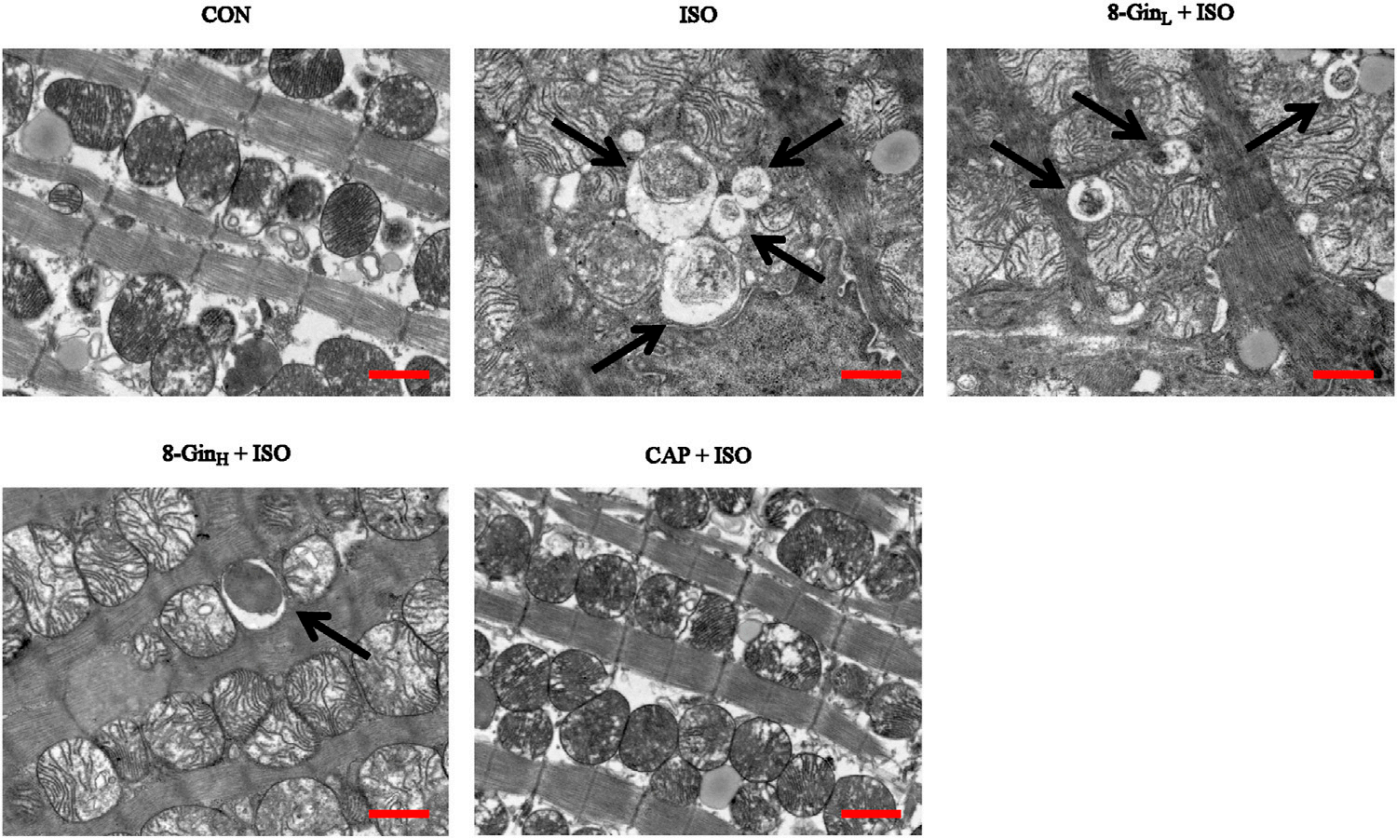
Effects of 8-Gin on the formation of autophagosome in mice after MF. The number of autophagosomes were shown in myocardial tissues by TEM (indicated by arrow, scale bar = 500 nm).

The formation of autophagosomes was observed in mice myocardial tissue of five groups using TEM. In Figure 7, we found an obvious increase in autophagosomes formation in the ISO group when compared with the CON group. In contrast, the number of autophagosomes evidently declined in the 8-Gin_L_ + ISO, 8-Gin_H_ + ISO, and CAP + ISO groups.

### Effects of 8-Gingerol on the Phosphatidylinositol-3-Kinase/Protein Kinase B/Mammalian Target of Rapamycin Signaling Pathway and Matrix Metalloproteinases-9 protein

**FIGURE 8:**
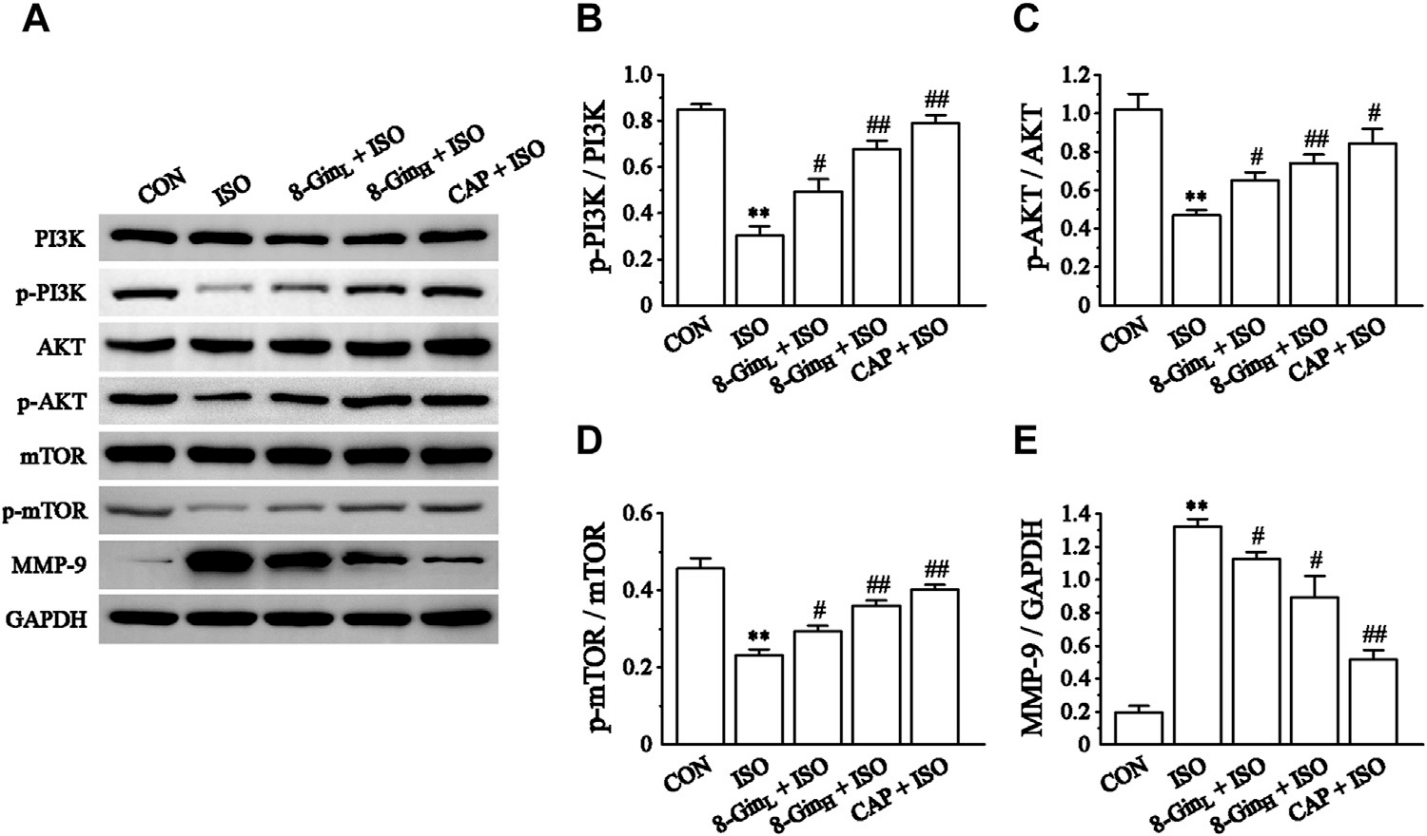
Effects of 8-Gin on the expression of the PI3K/AKT/mTOR signaling pathway relevant proteins and MMP-9 protein. **(A)** Representative images of PI3K/AKT/mTOR signaling pathway protein and MMP-9 protein expression were exhibited from cardiac tissue in the CON, ISO, 8-Gin_L_ + ISO, 8-Gin_H_ + ISO, and CAP + ISO groups. **(B–D)** Pooled analysis of PI3K, *p*-PI3K, AKT, p-AKT, mTOR, and p-mTOR protein expression by western blot analysis on which intensity was normalized to GAPDH. **(E)** Pooled analysis of MMP-9 protein expression by western blot analysis on which intensity was normalized to GAPDH. Data are shown as the mean ± SEM, *n* = 3. ^**^ *p* < 0.01 vs. the CON group; ^#^ *p* < 0.05, ^##^ *p* < 0.01 vs. the ISO group.

The changes in the PI3K/AKT/mTOR signaling pathway-related proteins and MMP-9 protein levels in mice hearts were detected using western blotting. In Figure 8, the expression of p-PI3K, p-AKT, p-mTOR, and MMP-9 proteins in the ISO group was down-regulated rapidly compared to the CON group (*p* < 0.01). However, after pretreatment with 8-Gin, the expression of both proteins were significantly up-regulated (*p* < 0.01 or 0.05).

### Effects of 8-Gingerol on the Proteins Expression of Caspase-9, Bax, and Bcl-2

**FIGURE 9:**
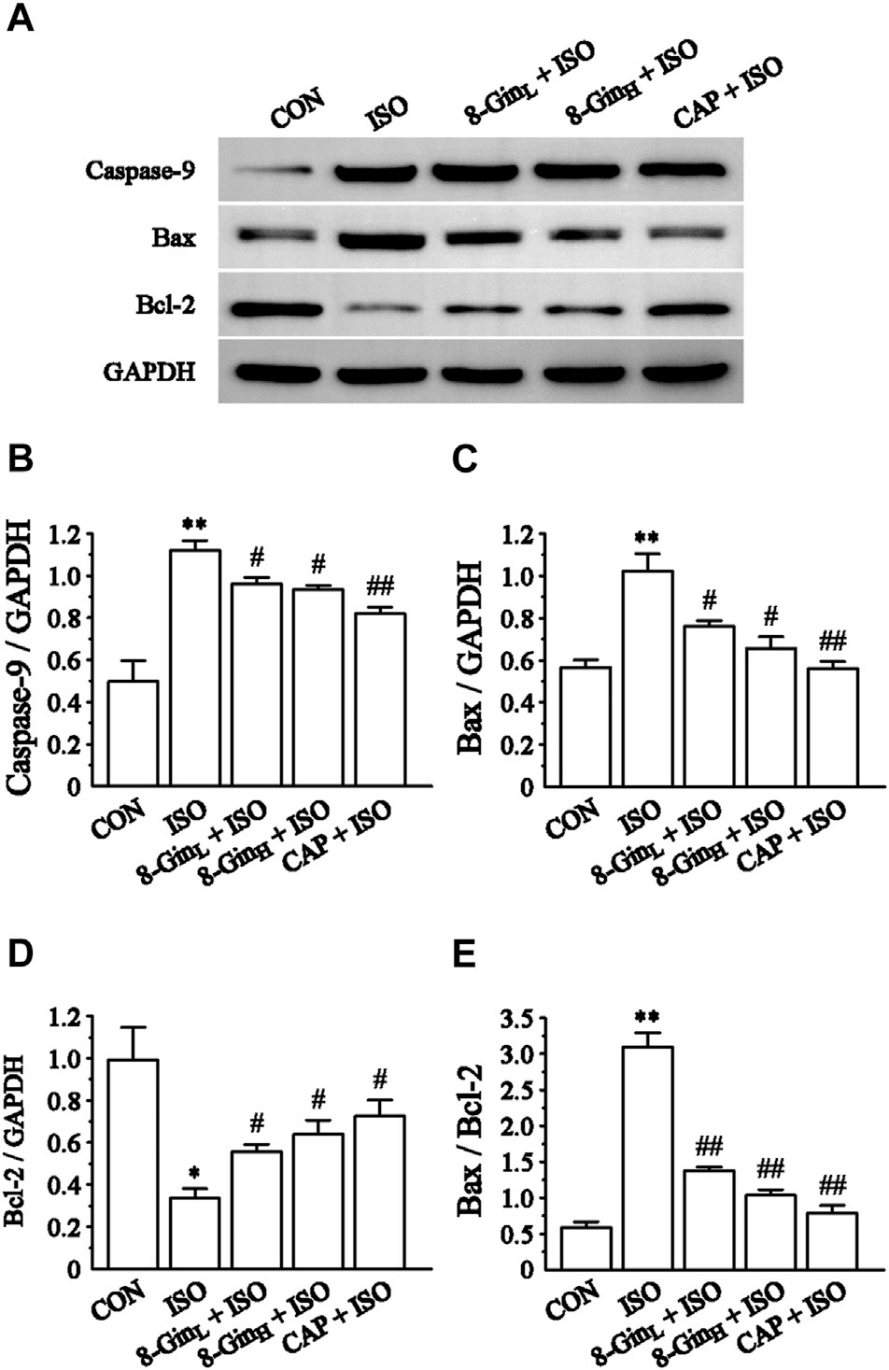
Effects of 8-Gin on the expression of Caspase-9, Bax, Bcl-2 protein. **(A)** Representative images of Caspase-9, Bax, Bcl-2 protein expression that were exhibited from cardiac tissue in the CON, ISO, 8-Gin_L_ + ISO, 8-Gin_H_ + ISO, and CAP + ISO groups. **(B–D)** Pooled analysis of Caspase-9, Bax, Bcl-2 protein expression by western blot analysis on which intensity was normalized to GAPDH. **(E)** The ratio of Bax and Bcl-2 protein expression by western blot analysis. Data are shown as the mean ± SEM, *n* = 3. ^*^ *p* < 0.05, ^**^ *p* < 0.01 vs. the CON group; ^#^ *p* < 0.05, ^##^ *p* < 0.01 vs. the ISO group.

Figures 9B,C showed that compared to the CON group, the levels of Caspase-9 and Bax protein markedly increased (*p* < 0.01), while the level of Bcl-2 protein decreased in the ISO group (*p* < 0.01 or 0.05). Moreover, as shown in Figure 9D, the ratio of Bax and Bcl-2 was also clearly up-regulated in the ISO group when matched with the CON group (*p* < 0.01). After pretreatment with 8-Gin, this result was reversed when compared with the ISO group (*p* < 0.01).

## Discussion

With the development of the aging population, the morbidity and mortality of heart disease is also increasing worldwide. MF is the most common pathological basis of multiple cardiovascular diseases. This pathology causes destruction of cardiac structures and eventually leads to into heart failure (Mao et al., 2019). Therefore, inhibition of MF is of great significance for improving cardiac structure and protecting cardiac function. In our present study, the MF model was established by subcutaneously injecting ISO into the mice and exploring the potential myocardial protection mechanisms of 8-Gin against ISO-induced MF. 8-Gin may exert beneficial actions through suppression of ROS, apoptosis, and excessive autophagy *via* the activation of the PI3K/Akt/mTOR signaling pathway.

ISO is a catecholamine, which can increase myocardial contractility, heart rate, conduction velocity, and myocardial oxygen consumption via excitation of the cardiac β1 receptor (Zick et al., 2008). Daily injections of a certain dose of ISO can cause a significant increase in the levels of plasma renin, aldosterone, and angiotensin II and an increase in the synthesis of myocardial interstitial collagen all of which contribute to development of MF (Dugasani et al., 2010; Lu et al., 2011). Therefore, ISO has been widely used for inducing MF in various models. Our results indicate that when compared with the CON group, heart rate and J-point elevation significantly increased in the ISO group (Figures 2B,C). In addition, after subcutaneous injection of ISO, the mice HW, CWI, and LVWI were also evidently increased (Table 1). Previous researches demonstrated that the increase of HW may be related to the increase of myocardial interstitial water volume caused by edema (Upaganlawar et al., 2009; Raish et al., 2019). It is widely known that CK and LDH were used as biochemical markers for detection of the degree of myocardial injury (Kobayashi et al., 1988). Our present study found that CK and LDH levels in the serum were markedly increased in the ISO group (Figures 3A,B). Besides, the results of histopathology suggested that ISO could induce myocardial disarray, myocardial interstitial edema, cardiac fibroblasts proliferation, and myocardial cell apoptosis (Figure 3). Based on the above results, it was concluded that we had successfully established an MF model in the mice. In contrast, it was observed that the ISO-induced MF was reversed after treatment with 8-Gin as indicated by the reduction in heart rate, J-point elevation, HW, CWI, LVWI, and CK and LDH levels and the improvement in histomorphology in the mice heart.

Under normal physiological conditions, the synthesis and degradation of the extracellular matrix (ECM) in the normal heart maintains a dynamic balance (Ma et al., 2018; Han et al., 2020). Previous study demonstrated that an imbalance between ECM synthesis and degradation may induce MF (Zhang et al., 2017b). The ECM is composed mainly of collagen types I and III (85 and 11%, respectively), which are regarded as biomarkers of MF (Chu et al., 2020; Hassan et al., 2020). TGF-β, a pro-fibrosis factor, plays an important regulatory role in the development of MF. It can be activated by ROS and inflammatory factors, which promotes the occurrence of fibrosis (Khalil et al., 2017). TGF-β can stimulate the proliferation and differentiation of myocardial fibroblasts, promote the expression of collagen type I and III, meanwhile, TGF-β can inhibit proteolytic enzyme, and finally accelerate the deposition of ECM and lead to MF (Khan and Sheppard, 2006). Matrix metalloproteinases (MMPs) are members of a zinc-dependent endopeptidase family. Studies have considered that MMP-9 plays a key role in ECM remodeling and the occurrence and development of MF (Li et al., 2013; Chen et al., 2015). In the early stages of fibrosis, MMP-9 is the major enzyme causing ECM decomposition, which can lead to ECM degeneration, a disorder of myocardial arrangement and abnormal systolic function (Wu et al., 2018). Studies have found that sustained activation of β-adrenoceptors leads to an increase in the synthesis and secretion of fibrillar collagen types I and III and finally to pathological MF (Matboli et al., 2019). Our present data showed the concentration of TGF-β markedly increased in the ISO group comparing to the CON group. On the contrary, TGF-β contents were reduced after treatment with 8-Gin (Figure 4B). Sirius red staining and ELISA quantitative analysis showed that when compared with the CON group, the accumulation of collagen types I and III significantly increased in the ISO group (Figures 4C,D). However, treatment with 8-Gin inhibited the excessive production of both collagen types I and III. Besides, MMPs also participate in the development of MF. Wang et al. (2019a) reported MF was associated with increased deposition of MMPs in the myocardium. Recent research has suggested that up-regulated MMP-9 may promote fibrosis by activating transforming growth factor beta (TGF-β) signaling. Moreover, several studies have indicated that MMP-9 is involved in the transformation of ECM and fibrosis and that it also promotes vascular smooth muscle cell proliferation (Krantz et al., 2011; Wang et al., 2019b). In our research, we found that 8-Gin caused a marked down-regulation of the expression of MMP-9 (Figure 8E), which suggests that 8-Gin could be effective against ISO-induced MF. However, MF is a complex pathological process, and the main characteristic pathological change is transdifferentiation of cardiac fibroblasts into myofibroblasts, which is the key and core of the formation of cardiac remodeling (Weber et al., 2013). Meanwhile, many studies have found that ISO also induced the occurrence of cardiac remodeling besides MF (Parthasarathy et al., 2014; Shanmugam et al., 2019). Therefore, we speculated that the 8-Gin may be also proposed for the treatment of tissue remodeling following an ischemic event.

In recent years, ROS has been recognized as a key factor in MF formation and development. Studies have shown that ROS plays an important role in cardiac fibroblast proliferation, collagen synthesis, and ECM metabolism (Suh et al., 2009). ROS can regulate the proliferation of the fibroblasts and promote MF by increasing the degradation of ECM proteins and activating MMP-9 expression (Suh et al., 2006). In addition, mounting evidence has revealed that overproduced ROS may play an important role in the process of excessive autophagy resulting in cell apoptosis (Forman et al., 2002). TEM is perhaps one of the most rigorous methods for clearly observing the process of ongoing mitochondrial autophagy. The PI3K/AKT/mTOR signaling pathway is considered to be a major signaling pathway regulating autophagy and apoptosis. The PI3K/AKT/mTOR signaling pathway has been shown to negatively regulate autophagy (Lassegue and Griendling, 2004). A previous study has explored activation of the PI3K/AKT/mTOR pathway in excessive autophagy inhibition (Butler et al., 2017). The Bcl-2 protein family plays a key role in cell apoptosis. Factors, such as starvation, oxygen deficiency, and ROS were shown to cause an inhibition of the anti-apoptotic protein Bcl-2 in contrast to activating the pro-apoptotic effector Bax (Wang et al., 2020). This condition leads to the release of cytochrome c, sensitization of Caspase-9 expression, thus contributing to activation of the effector Caspase-3 and finally resulting in cell apoptosis (Jiang et al., 2017b). Additionally, Nieuwenhuis *et al.* discovered that Bcl-2 and Caspase family proteins and the activation of PI3K/AKT signaling pathway are both involved in anti-apoptotic effects in human fibroblasts (Zhu et al., 2019). In the present study, we found the generation of ROS multiplied in the ISO group in contrast with the CON group, but 8-Gin significantly reduced ROS production (Figure 5). Also, we discovered that 8-Gin could cause a distinct up-regulation of the protein expression of p-PI3K, p-AKT, and p-mTOR (Figures 8B–D). According to the TEM results, we arrived at the conclusion that an increase in autophagosomes formation had occurred in the ISO group (Figure 7). Compared to the ISO group, the 8-Gin group had a smaller number of autophagosomes. TUNEL results implied that 8-Gin led to a marked reduction in the positive area of cardiomyocyte apoptosis (Figure 6). Furthermore, after treatment with 8-Gin, Caspase-9 and Bax protein expressions and the ratio of Bax and Bcl-2 were significantly down-regulated, while Bcl-2 protein expression was up-regulated compared to the ISO group (Figures 9B–D). These results indicated that 8-Gin could activate the PI3K/AKT/mTOR signaling pathway, which suppressed excessive production of autophagy and inhibits cardiomyocyte apoptosis.

CAP, an angiotensin-converting enzyme inhibitor commonly used in clinic, has been reported that is effective at ameliorating MF (Abareshi et al., 2017; Wang et al., 2019c), so it was used as a positive control in this study. According to our present results, 8-Gin produced an anti-fibrotic effect similar to that of CAP, but the therapeutic effect of CAP was stronger than 8-Gin. CAP is a chemical synthetic drug, while 8-Gin is a natural product extracted from plant ginger used as a condiment in our daily life. Therefore, compared with CAP, 8-Gin may have the characteristics of more safety, less adverse reactions and simpler production process.

Our results were based on *in vivo* experiments. However, in contrast with *in vivo* experiments, *in vitro* experiments can achieve species-specific, simpler, more convenient and detailed analysis. Thus, further research is needed to verify our results. In addition, the metabolic pathways of 8-Gin and a better characterization of the cell death mechanisms involved will require further investigation.

## Conclusion

In summary, our research investigated the cardio-protective effects and potential mechanisms of 8-Gin on ISO-induced MF. The study results demonstrate that 8-Gin caused a reduction in excessive ROS generation followed by activation of the PI3K/AKT/mTOR signaling pathway, a decrease in autophagosomes formation in order to suppress excessive autophagy, and eventually inhibited cardiomyocyte apoptosis. Based on our data, it appears that 8-Gin may become a candidate drug for the clinical therapy of MF. However, its clinical utility and generalized application remain to be further investigated.

## Data Availability

The data analyzed in this study is subject to the following licenses/restrictions: The datasets generated during and/or analysed during the current study are available from the corresponding author on reasonable request. Requests to access these datasets should be directed to LC, chuli0614@126.com.

## References

[B1] Abareshi A. Norouzi F. Asgharzadeh F. Beheshti F. Hosseini M. Farzadnia M. (2017). Effect of Angiotensin-Converting Enzyme Inhibitor on Cardiac Fibrosis and Oxidative Stress Status in Lipopolysaccharide-Induced Inflammation Model in Rats. Int. J. Prev. Med. 8, 69. 10.4103/ijpvm.IJPVM_322_16 28966758 PMC5609356

[B2] Bittencourt M. I. Cader S. A. Araújo D. V. Salles A. L. F. Albuquerque F. N. d. Spineti P. P. d. M. (2019). Role of Myocardial Fibrosis in Hypertrophic Cardiomyopathy: A Systematic Review and Updated Meta-Analysis of Risk Markers for Sudden Death. Arq. Bras. Cardiol. 112, 281–289. 10.5935/abc.20190045 30916191 PMC6424049

[B3] Butler D. E. Marlein C. Walker H. F. Frame F. M. Mann V. M. Simms M. S. (2017). Inhibition of the PI3K/AKT/mTOR Pathway Activates Autophagy and Compensatory Ras/Raf/MEK/ERK Signalling in Prostate Cancer. Oncotarget 8, 56698–56713. 10.18632/oncotarget.18082 28915623 PMC5593594

[B4] Cabral-Pacheco G. A. Garza-Veloz I. Castruita-De la Rosa C. Ramirez-Acuña J. M. Perez-Romero B. A. Guerrero-Rodriguez J. F. (2020). The Roles of Matrix Metalloproteinases and Their Inhibitors in Human Diseases. Ijms 21, 9739. 10.3390/ijms21249739 PMC7767220 33419373

[B5] Chen H. Xu Y. Wang J. Zhao W. Ruan H. (2015). Baicalin Ameliorates Isoproterenol-Induced Acute Myocardial Infarction through iNOS, Inflammation and Oxidative Stress in Rat. Int. J. Clin. Exp. Pathol. 8, 10139–10147. 26617721 PMC4637536

[B6] Chen S. Guo D. Lei B. Bi J. Yang H. (2020). Biglycan Protects Human Neuroblastoma Cells from Nitric Oxide-Induced Death by Inhibiting AMPK-mTOR Mediated Autophagy and Intracellular ROS Level. Biotechnol. Lett. 42, 657–668. 10.1007/s10529-020-02818-z 31989342

[B7] Chiao Y. A. Ramirez T. A. Zamilpa R. Okoronkwo S. M. Dai Q. Zhang J. (2012). Matrix Metalloproteinase-9 Deletion Attenuates Myocardial Fibrosis and Diastolic Dysfunction in Ageing Mice. Cardiovasc. Res. 96, 444–455. 10.1093/cvr/cvs275 22918978 PMC3500048

[B8] Chu X. Zhang Y. Xue Y. Li Z. Shi J. Wang H. (2020). Crocin Protects against Cardiotoxicity Induced by Doxorubicin through TLR-2/nf-κB Signal Pathway *in vivo* and Vitro. Int. Immunopharmacology 84, 106548. 10.1016/j.intimp.2020.106548 32388215

[B9] Dadakhujaev S. Jung E. J. Noh H. S. Hah Y.-S. Kim C. J. Kim D. R. (2009). Interplay between Autophagy and Apoptosis in TrkA-Induced Cell Death. Autophagy 5, 103–105. 10.4161/auto.5.1.7276 19115484

[B10] de Jong S. van Veen T. A. B. de Bakker J. M. T. Vos M. A. van Rijen H. V. M. (2011). Biomarkers of Myocardial Fibrosis. J. Cardiovasc. Pharmacol. 57, 522–535. 10.1097/FJC.0b013e31821823d9 21423029

[B11] Dugasani S. Pichika M. R. Nadarajah V. D. Balijepalli M. K. Tandra S. Korlakunta J. N. (2010). Comparative Antioxidant and Anti-inflammatory Effects of [6]-gingerol, [8]-gingerol, [10]-gingerol and [6]-shogaol. J. Ethnopharmacology 127, 515–520. 10.1016/j.jep.2009.10.004 19833188

[B12] Eguchi M. Kim Y. H. Kang K. W. Shim C. Y. Jang Y. Dorval T. (2012). Ischemia-Reperfusion Injury Leads to Distinct Temporal Cardiac Remodeling in Normal versus Diabetic Mice. PLoS One 7, e30450. 10.1371/journal.pone.0030450 22347376 PMC3275560

[B13] Ekor M. (2014). The Growing Use of Herbal Medicines: Issues Relating to Adverse Reactions and Challenges in Monitoring Safety. Front. Pharmacol. 4, 177. 10.3389/fphar.2013.00177 24454289 PMC3887317

[B14] Filomeni G. De Zio D. Cecconi F. (2015). Oxidative Stress and Autophagy: the Clash between Damage and Metabolic Needs. Cell Death Differ 22, 377–388. 10.1038/cdd.2014.150 25257172 PMC4326572

[B15] Forman H. J. Torres M. Fukuto J. (2002). Redox Signaling. Mol. Cell. Biochem. 234/235, 49–62. 10.1023/a:1015913229650 12162460

[B16] Gatica D. Chiong M. Lavandero S. Klionsky D. J. (2015). Molecular Mechanisms of Autophagy in the Cardiovascular System. Circ. Res. 116, 456–467. 10.1161/CIRCRESAHA.114.303788 25634969 PMC4313620

[B17] Hale A. N. Ledbetter D. J. Gawriluk T. R. Rucker, III E. B. 3rd. (2013). Autophagy. Autophagy 9, 951–972. 10.4161/auto.24273 24121596 PMC3722331

[B18] Han X. Liu P. Liu M. Wei Z. Fan S. Wang X. (2020). [6]‐Gingerol Ameliorates ISO‐Induced Myocardial Fibrosis by Reducing Oxidative Stress, Inflammation, and Apoptosis through Inhibition of TLR4/MAPKs/NF‐κB Pathway. Mol. Nutr. Food Res. 64, 2000003. 10.1002/mnfr.202000003 32438504

[B19] Hassan S. Barrett C. J. Crossman D. J. (2020). Imaging Tools for Assessment of Myocardial Fibrosis in Humans: the Need for Greater Detail. Biophys. Rev. 12, 969–987. 10.1007/s12551-020-00738-w 32705483 PMC7429810

[B20] Hu N. Yang L. Dong M. Ren J. Zhang Y. (2015). Deficiency in Adiponectin Exaggerates Cigarette Smoking Exposure-Induced Cardiac Contractile Dysfunction: Role of Autophagy. Pharmacol. Res. 100, 175–189. 10.1016/j.phrs.2015.08.005 26276084

[B21] Jiang S. Wang Q. Feng M. Li J. Guan Z. An D. (2017a). C2-ceramide Enhances Sorafenib-Induced Caspase-dependent Apoptosis via PI3K/AKT/mTOR and Erk Signaling Pathways in HCC Cells. Appl. Microbiol. Biotechnol. 101, 1535–1546. 10.1007/s00253-016-7930-9 27807662

[B22] Jiang W. Chen Y. Li B. Gao S. (2017b). DBA-induced Caspase-3-dependent Apoptosis Occurs through Mitochondrial Translocation of Cyt-C in the Rat Hippocampus. Mol. Biosyst. 13, 1863–1873. 10.1039/c7mb00246g 28731097

[B23] Kang L.-L. Zhang D.-M. Jiao R.-Q. Pan S.-M. Zhao X.-J. Zheng Y.-J. (2019). Pterostilbene Attenuates Fructose-Induced Myocardial Fibrosis by Inhibiting ROS-Driven Pitx2c/miR-15b Pathway. Oxidative Med. Cell Longevity 2019, 1–25. 10.1155/2019/1243215 PMC6913258 31871537

[B24] Khalil H. Kanisicak O. Prasad V. Correll R. N. Fu X. Schips T. (2017). Fibroblast-specific TGF-β-Smad2/3 Signaling Underlies Cardiac Fibrosis. J. Clin. Invest. 127, 3770–3783. 10.1172/JCI94753 28891814 PMC5617658

[B25] Khan R. Sheppard R. (2006). Fibrosis in Heart Disease: Understanding the Role of Transforming Growth Factor-Beta1 in Cardiomyopathy, Valvular Disease and Arrhythmia. Immunology 118, 10–24. 10.1111/j.1365-2567.2006.02336.x 16630019 PMC1782267

[B26] Kobayashi M. Ishida Y. Shoji N. Ohizumi Y. (1988). Cardiotonic Action of [8]-gingerol, an Activator of the Ca++-Pumping Adenosine Triphosphatase of Sarcoplasmic Reticulum, in guinea Pig Atrial Muscle. J. Pharmacol. Exp. Ther. 246, 667–673. 2457078

[B27] Kong P. Christia P. Frangogiannis N. G. (2014). The Pathogenesis of Cardiac Fibrosis. Cell. Mol. Life Sci. 71, 549–574. 10.1007/s00018-013-1349-6 23649149 PMC3769482

[B28] Krantz S. B. Shields M. A. Dangi-Garimella S. Cheon E. C. Barron M. R. Hwang R. F. (2011). MT1-MMP Cooperates with KrasG12D to Promote Pancreatic Fibrosis through Increased TGF-β Signaling. Mol. Cancer Res. 9, 1294–1304. 10.1158/1541-7786.MCR-11-0023 21856775 PMC3196812

[B29] Lassegue B. Griendling K. K. (2004). Reactive Oxygen Species in hypertension*1An Update. Am. J. Hypertens. 17, 852–860. 10.1016/j.amjhyper.2004.02.004 15363831

[B30] Li L. Zhao Q. Kong W. (2018a). Extracellular Matrix Remodeling and Cardiac Fibrosis. Matrix Biol. 68-69, 490–506. 10.1016/j.matbio.2018.01.013 29371055

[B31] Li M. Jiang Y. Jing W. Sun B. Miao C. Ren L. (2013). Quercetin Provides Greater Cardioprotective Effect Than its Glycoside Derivative Rutin on Isoproterenol-Induced Cardiac Fibrosis in the Rat. Can. J. Physiol. Pharmacol. 91, 951–959. 10.1139/cjpp-2012-0432 24117263

[B32] Li M. Yang G. Xie B. Babu K. Huang C. (2014). Changes in Matrix Metalloproteinase-9 Levels during Progression of Atrial Fibrillation. J. Int. Med. Res. 42, 224–230. 10.1177/0300060513488514 24345823

[B33] Li X. Hu X. Wang J. Xu W. Yi C. Ma R. (2018b). Inhibition of Autophagy via Activation of PI3K/Akt/mTOR Pathway Contributes to the Protection of Hesperidin against Myocardial Ischemia/Reperfusion Injury. Int. J. Mol. Med. 42, 1917–1924. 10.3892/ijmm.2018.3794 30066841 PMC6108872

[B34] Lu C. Yang Y. Zhu Y. Lv S. Zhang J. (2018). An Intervention Target for Myocardial Fibrosis: Autophagy. Biomed. Res. Int. 2018, 1–10. 10.1155/2018/6215916 PMC5911341 29850542

[B35] Lu J. Guan S. Shen X. Qian W. Huang G. Deng X. (2011). Immunosuppressive Activity of 8-gingerol on Immune Responses in Mice. Molecules 16, 2636–2645. 10.3390/molecules16032636 21441866 PMC6259933

[B36] Ma D. Zhang J. Zhang Y. Zhang X. Han X. Song T. (2018). Inhibition of Myocardial Hypertrophy by Magnesium Isoglycyrrhizinate through the TLR4/NF-κB Signaling Pathway in Mice. Int. Immunopharmacology 55, 237–244. 10.1016/j.intimp.2017.12.019 29274625

[B37] Mao Q.-Q. Xu X.-Y. Cao S.-Y. Gan R.-Y. Corke H. Beta T. (2019). Bioactive Compounds and Bioactivities of Ginger (*Zingiber Officinale* Roscoe). Foods 8, 185. 10.3390/foods8060185 PMC6616534 31151279

[B38] Matboli M. Shafei A. E. Agwa S. H. A. Elzahy S. S. Anwar A. K. Mansour A. R. (2019). Identification of Novel Molecular Network Expression in Acute Myocardial Infarction. Cg 20, 340–348. 10.2174/1389202920666190820142043 PMC7235391 32476991

[B39] Medugorac I. Jacob R. (1983). Characterisation of Left Ventricular Collagen in the Rat. Cardiovasc. Res. 17, 15–21. 10.1093/cvr/17.1.15 6221797

[B40] Nieuwenhuis B. Lüth A. Kleuser B. (2010). Dexamethasone Protects Human Fibroblasts from Apoptosis via an S1P3-Receptor Subtype Dependent Activation of PKB/Akt and BclXL. Pharmacol. Res. 61, 449–459. 10.1016/j.phrs.2009.12.005 20005955

[B41] Parthasarathy A. Gopi V. Devi Km S. Balaji N. Vellaichamy E. (2014). Aminoguanidine Inhibits Ventricular Fibrosis and Remodeling Process in Isoproterenol-Induced Hypertrophied Rat Hearts by Suppressing ROS and MMPs. Life Sci. 118, 15–26. 10.1016/j.lfs.2014.09.030 25445437

[B42] Piek A. Silljé H. H. W. de Boer R. A. (2019). The Vicious Cycle of Arrhythmia and Myocardial Fibrosis. Eur. J. Heart Fail. 21, 492–494. 10.1002/ejhf.1421 30698320

[B43] Raish M. Ahmad A. Ansari M. A. Alkharfy K. M. Ahad A. Khan A. (2019). Beetroot Juice Alleviates Isoproterenol-Induced Myocardial Damage by Reducing Oxidative Stress, Inflammation, and Apoptosis in Rats. 3 Biotech. 9, 147. 10.1007/s13205-019-1677-9 PMC6430824 30944794

[B44] Ryan T. D. Rothstein E. C. Aban I. Tallaj J. A. Husain A. Lucchesi P. A. (2007). Left Ventricular Eccentric Remodeling and Matrix Loss Are Mediated by Bradykinin and Precede Cardiomyocyte Elongation in Rats with Volume Overload. J. Am. Coll. Cardiol. 49, 811–821. 10.1016/j.jacc.2006.06.083 17306712

[B45] Schirone L. Forte M. Palmerio S. Yee D. Nocella C. Angelini F. (2017). A Review of the Molecular Mechanisms Underlying the Development and Progression of Cardiac Remodeling. Oxidative Med. Cell Longevity 2017, 1–16. 10.1155/2017/3920195 PMC5511646 28751931

[B46] Shanmugam G. Challa A. K. Litovsky S. H. Devarajan A. Wang D. Jones D. P. (2019). Enhanced Keap1-Nrf2 Signaling Protects the Myocardium from Isoproterenol-Induced Pathological Remodeling in Mice. Redox Biol. 27, 101212. 10.1016/j.redox.2019.101212 31155513 PMC6859568

[B47] Suh S.-J. Jin U.-H. Kim S.-H. Chang H.-W. Son J.-K. Ho Lee S. (2006). Ochnaflavone Inhibits TNF-α-Induced Human VSMC Proliferation via Regulation of Cell Cycle, ERK1/2, and MMP-9. J. Cell. Biochem. 99, 1298–1307. 10.1002/jcb.20912 16795041

[B48] Suh S.-J. Kim J.-R. Jin U.-H. Choi H.-S. Chang Y.-C. Lee Y.-C. (2009). Deoxypodophyllotoxin, Flavolignan, from Anthriscus Sylvestris Hoffm. Inhibits Migration and MMP-9 via MAPK Pathways in TNF-α-Induced HASMC. Vasc. Pharmacol. 51, 13–20. 10.1016/j.vph.2008.10.004 19013539

[B49] Takino J.-i. Sato T. Nagamine K. Hori T. (2019). The Inhibition of Bax Activation-Induced Apoptosis by RasGRP2 via R-Ras-PI3K-Akt Signaling Pathway in the Endothelial Cells. Sci. Rep. 9, 16717. 10.1038/s41598-019-53419-4 31723205 PMC6854084

[B50] Tang Y. Jacobi A. Vater C. Zou L. Zou X. Stiehler M. (2015). Icariin Promotes Angiogenic Differentiation and Prevents Oxidative Stress-Induced Autophagy in Endothelial Progenitor Cells. Stem Cell 33, 1863–1877. 10.1002/stem.2005 25787271

[B51] Tayebjee M. H. MacFadyen R. J. Lip G. Y. (2003). Extracellular Matrix Biology. J. Hypertens. 21, 2211–2218. 10.1097/01.hjh.0000098178.36890.81 14654734

[B52] Upaganlawar A. Gandhi C. Balaraman R. (2009). Effect of Green Tea and Vitamin E Combination in Isoproterenol Induced Myocardial Infarction in Rats. Plant Foods Hum. Nutr. 64, 75–80. 10.1007/s11130-008-0105-9 19058010

[B53] Wang J. Shen W. Zhang J.-Y. Jia C.-H. Xie M.-L. (2019a). Stevioside Attenuates Isoproterenol-Induced Mouse Myocardial Fibrosis through Inhibition of the Myocardial NF-κB/TGF-β1/Smad Signaling Pathway. Food Funct. 10, 1179–1190. 10.1039/c8fo01663a 30735218

[B54] Wang L. Yuan D. Zheng J. Wu X. Wang J. Liu X. (2019b). Chikusetsu Saponin IVa Attenuates Isoprenaline-Induced Myocardial Fibrosis in Mice through Activation Autophagy Mediated by AMPK/mTOR/ULK1 Signaling. Phytomedicine 58, 152764. 10.1016/j.phymed.2018.11.024 31005723

[B55] Wang Q.-w. Yu X.-f. Xu H.-l. Zhao X.-z. Sui D.-y. (2019c). Ginsenoside Re Improves Isoproterenol-Induced Myocardial Fibrosis and Heart Failure in Rats. Evidence-Based Complement. Altern. Med. 2019, 1–9. 10.1155/2019/3714508 PMC6332977 30713572

[B56] Wang S.-Y. Zhao J.-M. Zhou C.-L. Zheng H.-D. Huang Y. Zhao M. (2020). Herbal Cake-Partitioned Moxibustion Inhibits Colonic Autophagy in Crohn's Disease via Signaling Involving Distinct Classes of Phosphatidylinositol 3-kinases. Wjg 26, 5997–6014. 10.3748/wjg.v26.i39.5997 33132650 PMC7584057

[B57] Weber K. T. Sun Y. Bhattacharya S. K. Ahokas R. A. Gerling I. C. (2013). Myofibroblast-Mediated Mechanisms of Pathological Remodelling of the Heart. Nat. Rev. Cardiol. 10, 15–26. 10.1038/nrcardio.2012.158 23207731

[B58] Wu J. Bai Y. Wang Y. Ma J. (2021). Melatonin and Regulation of Autophagy: Mechanisms and Therapeutic Implications. Pharmacol. Res. 163, 105279. 10.1016/j.phrs.2020.105279 33161138

[B59] Wu X. Li M. Chen S. Q. Li S. Guo F. (2018). Pin1 Facilitates Isoproterenol-induced Cardiac Fibrosis and Collagen Deposition by Promoting Oxidative Stress and Activating the MEK1/2ERK1/2 Signal Transduction Pathway in Rats. Int. J. Mol. Med. 41, 1573–1583. 10.3892/ijmm.2017.3354 29286102 PMC5819929

[B60] Wymann M. P. Zvelebil M. Laffargue M. (2003). Phosphoinositide 3-kinase Signalling - Which Way to Target? Trends Pharmacol. Sci. 24, 366–376. 10.1016/S0165-6147(03)00163-9 12871670

[B61] Zhang F. Ma N. Gao Y.-F. Sun L.-L. Zhang J.-G. (2017a). Therapeutic Effects of 6-Gingerol, 8-Gingerol, and 10-Gingerol on Dextran Sulfate Sodium-Induced Acute Ulcerative Colitis in Rats. Phytother. Res. 31, 1427–1432. 10.1002/ptr.5871 28762585

[B62] Zhang N. Wei W.-Y. Li L.-L. Hu C. Tang Q.-Z. (2018). Therapeutic Potential of Polyphenols in Cardiac Fibrosis. Front. Pharmacol. 9, 122. 10.3389/fphar.2018.00122 29497382 PMC5818417

[B63] Zhang Y. Xie C. Li A. Liu X. Xing Y. Shen J. (2019). PKI-587 Enhances Chemosensitivity of Oxaliplatin in Hepatocellular Carcinoma through Suppressing DNA Damage Repair Pathway (NHEJ and HR) and PI3K/AKT/mTOR Pathway. Am. J. Transl. Res. 11, 5134–5149. 31497229 PMC6731445

[B64] Zhang Z. Yang C. Shen M. Yang M. Jin Z. Ding L. (2017b). Autophagy Mediates the Beneficial Effect of Hypoxic Preconditioning on Bone Marrow Mesenchymal Stem Cells for the Therapy of Myocardial Infarction. Stem Cel Res. Ther. 8, 89. 10.1186/s13287-017-0543-0 PMC5395756 28420436

[B65] Zhao Q. Li H. Chang L. Wei C. Yin Y. Bei H. (2019). Qiliqiangxin Attenuates Oxidative Stress-Induced Mitochondrion-dependent Apoptosis in Cardiomyocytes via PI3K/AKT/GSK3β Signaling Pathway. Biol. Pharm. Bull. 42, 1310–1321. 10.1248/bpb.b19-00050 31142701

[B66] Zhao Y. Feng X. Li B. Sha J. Wang C. Yang T. (2020). Dexmedetomidine Protects against Lipopolysaccharide-Induced Acute Kidney Injury by Enhancing Autophagy through Inhibition of the PI3K/AKT/mTOR Pathway. Front. Pharmacol. 11, 128. 10.3389/fphar.2020.00128 32158395 PMC7052304

[B67] Zhu A. Sun Y. Zhong Q. Yang J. Zhang T. Zhao J. (2019). Effect of Euphorbia Factor L1 on Oxidative Stress, Apoptosis, and Autophagy in Human Gastric Epithelial Cells. Phytomedicine 64, 152929. 10.1016/j.phymed.2019.152929 31454650

[B68] Zick S. M. Djuric Z. Ruffin M. T. Litzinger A. J. Normolle D. P. Alrawi S. (2008). Pharmacokinetics of 6-gingerol, 8-gingerol, 10-gingerol, and 6-shogaol and Conjugate Metabolites in Healthy Human Subjects. Cancer Epidemiol. Biomarkers Prev. 17, 1930–1936. 10.1158/1055-9965.EPI-07-2934 18708382 PMC2676573

